# Optical coherence tomography guided targeted non-viral optical delivery of opsin-encoding genes into the retina

**DOI:** 10.3389/fphar.2026.1831339

**Published:** 2026-10-19

**Authors:** Sanghoon Kim, Michael Carlson, Subrata Batabyal, Adnan Dibas, Samarendra Mohanty

**Affiliations:** 1 Nanoscope Technologies LLC., Bedford, TX, United States; 2 Nanoscope Therapeutics, Inc., Dallas, TX, United States

**Keywords:** gene therapy, multi-characteristic opsin, non-viral delivery, optogenetics, retinal degeneration

## Abstract

**Introduction:**

Retinal degenerative diseases cause progressive photoreceptor loss while leaving populations of inner retinal neurons available for optogenetic intervention. However, efficient and spatially controlled non‐viral gene delivery to these surviving cells remains challenging. We evaluated optical coherence tomography (OCT)‐guided nano-enhanced optical delivery (NOD) for targeted delivery of the multi-characteristic opsin MCO‐020 to the degenerative retina.

**Methods:**

NOD uses plasmonic gold nanorods (GNRs) and a 980‐nm near‐infrared laser to transiently permeabilize cell membranes and facilitate localized plasmid delivery. Delivery parameters were initially optimized in HEK293 cells. *In vivo* efficacy and safety were subsequently evaluated in rd1 mice following intravitreal administration of MCO-020 plasmid and GNRs followed by OCT‐guided laser delivery. Transgene expression, retinal structure, retinal and cortical electrophysiological responses, intraocular pressure, retinal‐cell viability, and biodistribution were evaluated.

**Results:**

*In vitro* studies demonstrated laser‐dependent and spatially restricted MCO‐020 expression without detectable cytotoxicity within the tested operating range. *In vivo*, OCT‐guided NOD produced MCO‐020 expression in inner retinal cells within targeted retinal regions without detectable changes in retinal structure or thickness. MCO‐020‐treated mice demonstrated increased light‐evoked retinal and visually evoked cortical responses compared with untreated controls, with responses and transgene expression remaining detectable through the 52‐week observation period. Retinal‐cell viability, intraocular pressure, and complementary electrophysiological safety assessments showed no detectable treatment-associated adverse effects. Biodistribution analysis showed only low levels of vector DNA in evaluated non‐target tissues.

**Discussion:**

These findings demonstrate that OCT‐guided NOD can provide spatially targeted, non‐viral delivery of an opsin‐encoding plasmid to the degenerative retina while preserving retinal structure and supporting sustained functional engagement of the visual pathway. The approach may provide a platform for localized delivery of therapeutic genes to retinal regions affected by degeneration.

## Introduction

Retinal degenerative diseases represent a major cause of irreversible vision loss worldwide and are becoming increasingly prevalent with the aging population. These disorders are characterized by progressive degeneration of retinal cells, particularly photoreceptors and retinal pigment epithelium (RPE), leading to impairment of the visual transduction cascade. Severe vision loss occurs in several inherited and age-related retinal disorders, including dry age-related macular degeneration (dry AMD), retinitis pigmentosa (RP), Leber congenital amaurosis (LCA), and Stargardt disease (Hartong et al., 2006; Ferrari et al., 2011; Holz et al., 2007; Mittra and Singerman, 2002). In many of these conditions, although photoreceptors degenerate, higher-order retinal neurons such as bipolar cells and retinal ganglion cells remain preserved for extended periods. This preservation of inner retinal circuitry provides an opportunity to restore visual function by converting these surviving neurons to light-sensing cells (Sahel and Roska, 2013; Sahel et al., 2019; Busskamp et al., 2012).

Gene therapy has emerged as a promising strategy for treating retinal degenerative diseases by introducing functional healthy copies of defective genes or correcting the mutation(s) in defective genes in affected cells (Naldini, 2015; Kaufmann et al., 2013; Xiao-Jie et al., 2015). Successful clinical translation of gene therapy for RPE65-associated LCA has demonstrated the feasibility of genetic interventions for retinal diseases and has stimulated interest in developing gene-based therapies for other retinal degenerative conditions (Sahel et al., 2019; Ashtari et al., 2015). Most current ophthalmic gene therapy approaches rely on viral vectors, particularly adeno-associated viruses (AAVs), to deliver therapeutic genes to retinal cells (Boye et al., 2013; Gonçalves, 2005; Wang et al., 2011). While viral delivery systems have demonstrated high transduction efficiency, they present several limitations. Viral vectors have limited packaging capacity, typically less than 4.7 kb, which restricts the delivery of large therapeutic genes and emerging gene-editing tools such as CRISPR/Cas9 (Wu et al., 2010; Lai et al., 2010; Wiethoff and Middaugh, 2003). Additionally, viral vectors may induce inflammatory responses, immunological reactions, and potential genomic integration events that may compromise safety (Wang et al., 2011; Srivastava, 2016). Another major limitation of viral delivery is the lack of spatial control of gene expression following administration (Bordet and Behar-Cohen, 2019).

For retinal diseases characterized by localized degeneration, such as geographic atrophy in advanced AMD, the ability to deliver therapeutic genes to spatially targeted regions of the retina is particularly advantageous. Targeted delivery could allow treatment of degenerated retinal areas while minimizing off-target gene expression in healthy tissue. Therefore, there is a strong need to develop minimally invasive, non-viral gene delivery strategies capable of delivering therapeutic molecules of varying sizes to spatially defined regions of the retina (Al-Dosari and Gao, 2009; Jones et al., 2013). Optogenetic approaches have emerged as a promising strategy to restore visual responses in photoreceptor-degenerated retina by rendering surviving retinal neurons photosensitive (Sahel and Roska, 2013; Busskamp et al., 2012; Busskamp et al., 2010; Klapoetke et al., 2014). We have developed a broadband multi-characteristic opsin (MCO) that can be activated by ambient light levels to stimulate higher-order retinal neurons with fast kinetics and high dynamic range (Wright et al., 2017). Expression of MCO in retinal neurons enables light-induced depolarization of these cells, allowing transmission and processing of visual signals through the existing retinal circuitry and into the visual cortex. Sahel et al. reported partial recovery of visual function in a patient with advanced retinitis pigmentosa following AAV-mediated expression of ChrimsonR combined with light-stimulating goggles (Sahel et al., 2021). More recently, MCO-010 has been evaluated clinically in patients with advanced retinitis pigmentosa and Stargardt disease, supporting the translational potential of MCO-based optogenetic approaches (Mohanty et al., 2025; Lam et al., 2025).

Several physical approaches have been investigated to facilitate non-viral gene delivery, including electroporation, microinjection, and laser-mediated transfection (Al-Dosari and Gao, 2009; Mellott et al., 2013; Tirlapur and König, 2002). Compared with electroporation and nanoparticle-based approaches, plasmonic optoporation offers more spatially confined transfection, although delivery efficiency and durability vary across experimental models. Among these, laser-based delivery methods have attracted considerable attention because they enable highly localized and minimally invasive membrane permeabilization (Yao et al., 2008). To enable efficient and spatially controlled delivery of the MCO gene, we have developed a nano-enhanced optical delivery (NOD) method. The NOD technique utilizes a near-infrared (NIR) laser beam together with plasmonic nano-enhancers, specifically gold nanorods (GNRs), to facilitate targeted delivery of plasmids into retinal cells. Gold nanorods efficiently absorb NIR light tuned to the peak of their surface plasmon resonance and convert the optical energy into localized thermal energy. When irradiated with an NIR laser beam, the GNRs generate localized hotspots at the cell surface that transiently permeabilize the cell membrane, enabling entry of extracellular plasmids into the cell. This mechanism allows targeted delivery of MCO-020 plasmids to laser-irradiated cells while minimizing effects on surrounding tissue. The NOD method has previously been demonstrated in both *in vitro* and *in vivo* systems to enable spatially targeted transgene expression in laser-irradiated regions (Dhakal et al., 2015; Batabyal et al., 2021).

To further enhance the spatial precision of gene delivery, here, we present integration of the scanning NIR laser delivery system with optical coherence tomography (OCT) imaging. OCT provides depth-resolved high-resolution structural imaging of the retina and enables identification of retinal regions of interest for targeted gene delivery. OCT has also been successfully combined with multiple imaging and therapeutic modalities—including angiography, fluorescence imaging, and laser-based microsurgery—to provide real-time structural guidance during ophthalmic interventions (Kim et al., 2012; Carlson et al., 2022; Leitgeb and Baumann, 2018). Leveraging these capabilities, OCT-guided NOD enables precise localization and delivery of therapeutic genes to targeted retinal regions. *In vivo* studies were conducted in mouse models to evaluate the pharmacokinetics, safety, and biodistribution of the therapy. Efficient and stable expression of MCO-reporter proteins was observed in the retina following intravitreal injection of MCO-020 plasmids mixed with gold nanorods and subsequent NIR laser irradiation. Toxicology and biodistribution analyses demonstrated that the delivery method was well tolerated and that the transgene was not detected in non-target organs. Collectively, these findings support the development of OCT-guided NOD as a minimally invasive and spatially targeted non-viral gene therapy platform. The MCO-020 approach enables efficient photosensitization of higher-order retinal neurons in photoreceptor-degenerated retina and represents a promising therapeutic strategy for the treatment of atrophic lesion(s).

## Materials and methods

### Ethics statement

All animal experiments were conducted in accordance with the guidelines of the Nanoscope Technologies Institutional Animal Care and Use Committee (IACUC) and complied with all relevant institutional and federal regulations governing the ethical use of laboratory animals. Animals were housed in temperature- and humidity-controlled facilities under a 12-h light/12-h dark cycle with free access to food and water.

### Preparation of MCO-020 plasmids

The therapeutic payload used in this study was the MCO-020 plasmid which encodes an MCO designed to confer ambient light sensitivity to retinal neurons. The plasmid construct consists of a CAG promoter driving expression of the MCO-020 coding sequence, followed by a human growth hormone polyadenylation signal (hGH polyA). The expression cassette was cloned into a plasmid backbone to generate the MCO-020 plasmid. The overall plasmid construct is shown in Figure 1A.

**FIGURE 1:**
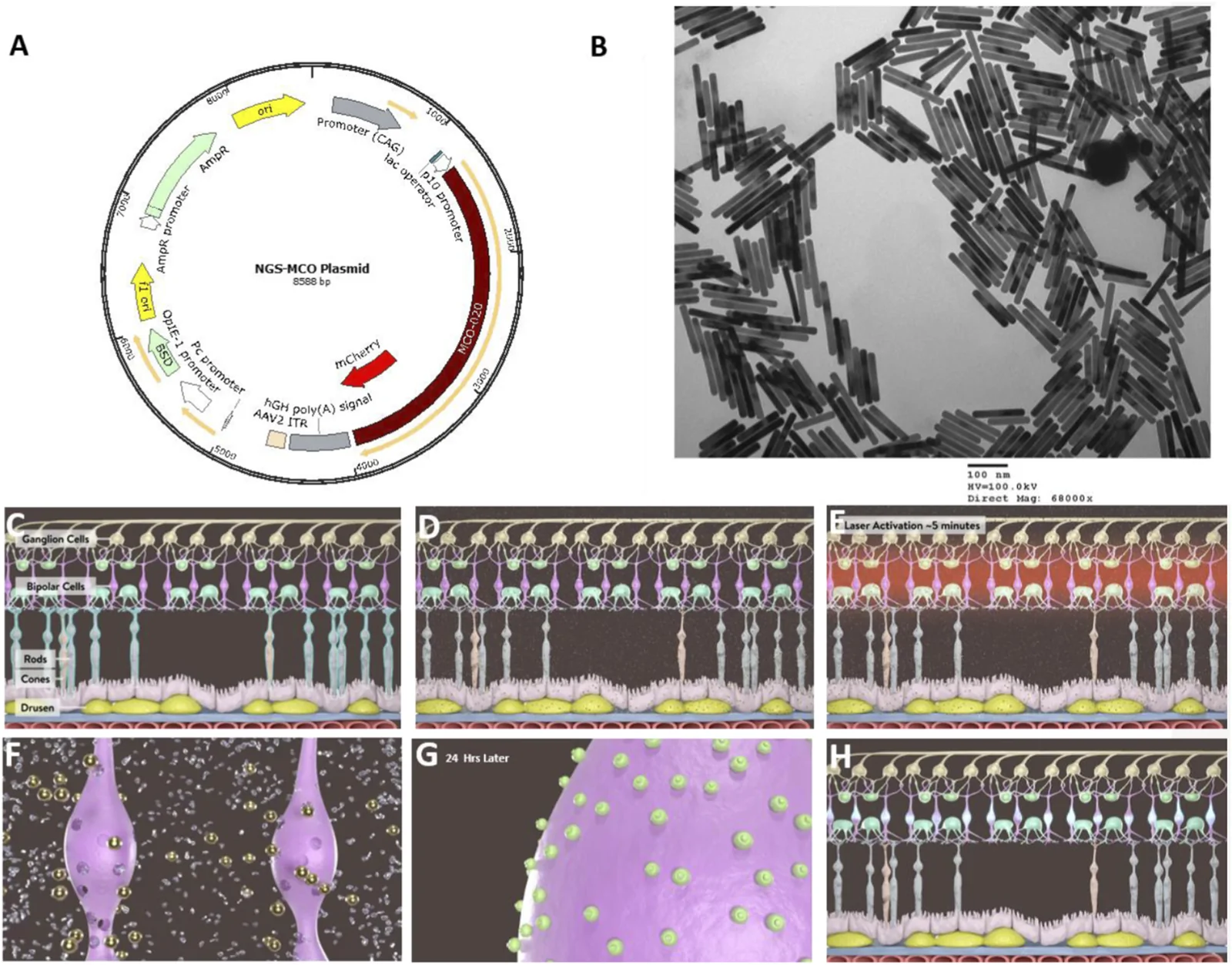
Schematic of the nano-enhanced optical delivery (NOD) process. **(A)** MCO-020 Plasmid construct map. The MCO-020 gene expression cassette comprises CAG promoter, MCO-020 transgene, and the human growth hormone polyadenylation site. **(B)** Transmission Electron Microscopic image of GNRs. To minimize non-specific binding, the GNRs are PEGylated. PEGylation also prevents the aggregation of the GNRs. **(C)** The geographic atrophic region where photoreceptor loss has occurred is depicted. **(D)** The GNR and MCO-020 plasmids are settled in the retinal layers after intravitreal injection. **(E)** The retinal layers are irradiated with near-infrared (NIR) light, guided by optical coherence tomography (OCT), to generate a hotspot (by GNRs) for targeted delivery of MCO-020 to cells. **(F)** The presence of PEGylated GNRs near the cell surface generates localized plasmonic heating of the cell membrane upon NIR light irradiation, transiently permeabilizing the membrane and allowing MCO-020 to enter the cells. **(G)** MCO-020 opsin is expressed in the cells, rendering them photosensitive and potentially restoring light sensitivity to the atrophic lesion. **(H)** An electrical signal propagates through higher-order neurons to the brain, resulting in the restoration of light sensitivity in the atrophic lesion.

Amplification and purification of the plasmid DNA were carried out using standard *E*. *coli* bacterial transformation and plasmid purification procedures using Zymo Pure (Zymo Research Corporation). The sample concentration and purity were measured by a nanophotometer (Implen). The integrity and identity of the plasmid construct were verified by restriction enzyme digestion followed by agarose gel electrophoresis.

### Gold nanorods

Gold nanorods (GNRs; Nanopartz Inc.) were used as plasmonic nano-enhancers for optical delivery. The GNRs were functionalized with 5 kDa carboxyl-terminated polyethylene glycol (PEG) and supplied as a sterile, endotoxin-purified formulation at a nominal concentration of 2.5 mg/mL and optical density of 50. Transmission electron microscopy (TEM) confirmed the rod-shaped morphology of the particles (Figure 1B). Analysis of 75 nanorods showed average dimensions of approximately 120 nm in length and 25 nm in width, with the corresponding size distributions shown in Supplementary Figure 1A,B. The GNR formulation had a zeta potential of approximately −28 mV at pH 7. UV–Vis–NIR characterization showed a strong longitudinal plasmonic absorption band in the near-infrared region overlapping the 980 nm wavelength used for NOD delivery (Supplementary Figure 1C). These physicochemical and optical properties supported use of the GNRs for 980 nm plasmonic optical delivery. The nanorods were PEGylated to minimize non-specific binding and to prevent nanoparticle aggregation in physiological solutions. PEGylation provided improved nanoparticle stability and reduced nonspecific interactions during *in vivo* delivery.

### Animal model of retinal degeneration

Retinal degeneration experiments were conducted using *rd1* mice (C3H/HeJ-Pde6brd1), which were obtained from the Jackson Laboratory and bred at the Nanoscope Technologies animal facility. The *rd1* mouse carries a mutation in the *Pde6b* gene encoding the β-subunit of rod photoreceptor phosphodiesterase. This mutation results in rapid degeneration of rod photoreceptors followed by secondary degeneration of cone photoreceptors, leading to severe visual impairment. Because photoreceptor cells are largely absent while inner retinal neurons remain relatively preserved, the *rd1* mouse provides a suitable model for evaluating optogenetic strategies aimed at restoring light sensitivity in the degenerative retina. Both male and female *rd1* mice were included in the study, with balanced representation of sexes across the principal *in vivo* treatment groups. Sample sizes for individual experiments are provided in the corresponding figure legends. One eye was assigned to receive the MCO-020/GNR formulation and OCT-guided NOD treatment. To further evaluate the therapeutic potential of the NOD delivery platform in localized retinal degeneration, a mouse model of laser injury was generated by inducing focal photoreceptor injury in wild-type mice using a green laser beam (532 nm, 50 mW). The *rd1* mouse model was used as proof-of-concept of severe photoreceptor degeneration for evaluating delivery to surviving inner retinal cells. Although this model provides rapid and extensive outer-retinal degeneration, it represents inherited retinal degeneration rather than age-related macular degeneration and does not reproduce the chronic RPE dysfunction, inflammatory processes, or macular anatomy associated with human geographic atrophy. A separate laser-injury model was used to generate a spatially confined region of retinal damage for evaluation of OCT-guided localized delivery of MCO-020. This acute injury model was not intended to reproduce the full pathogenesis of human geographic atrophy.

### Randomization and masking

A randomized experimental design was implemented to minimize experimental bias. Animals were randomly assigned to designated treatment groups before treatment, with balanced representation of male and female mice across the principal *in vivo* treatment groups. Cage numbers, group numbers, and animal identifiers were coded to ensure masking during imaging experiments, electrophysiological measurements and data analysis. Investigators performing imaging and data analysis were blinded to treatment allocation until completion of the analysis.

### Intraocular pressure measurement

Intraocular pressure (IOP) was measured using a rebound tonometer (Tonovet Plus, iCARE). Mice were allowed to rest briefly prior to measurement. The tonometer probe was positioned parallel to the eye and aligned with the center of the cornea. Each measurement consisted of multiple consecutive readings, and three trials were performed for each eye to yield an average value. The same instrument, operator, positioning procedure, and acquisition conditions were used for the treated and contralateral un-injected eyes at all time points.

### Electrophysiological measurements

Electrophysiological recordings were performed to evaluate retinal and cortical responses following treatment. Electroretinography (ERG) and visually evoked potential (VEP) recordings were acquired using the NS-NEEL electrophysiology acquisition system (Nanoscope Instruments). Mice were dark-adapted overnight prior to recordings. For electrophysiological measurements, animals were anesthetized using either isoflurane (ERG recordings) or an intraperitoneal mixture of ketamine (90 mg/kg), xylazine (10 mg/kg), and acepromazine (0.5 mg/kg) for VEP recordings. Pupils were dilated using topical 1% tropicamide. During recordings, animals were maintained on a temperature-controlled heating pad at approximately 35 °C, and the cornea was kept hydrated with balanced salt solution.

Electrical signals were amplified using a built-in bias drive amplifier and digitized through analog-to-digital converters equipped with a programmable gain amplifier. Data acquisition was performed at a sampling rate of 1 kHz. During acquisition, signals were filtered using a 60 Hz notch filter and a 0.1–300 Hz bandpass filter. For each stimulus condition, multiple responses were recorded and averaged to improve the signal-to-noise ratio. No normalization was applied to the raw data, and averaged waveforms were used for subsequent analyses.

### Measurement of electroretinography (ERG)

Scotopic ERG recordings were performed to evaluate retinal function. A ground electrode was inserted at the base of the tail, and a reference electrode was placed sub-dermally between the eyes. A silver-wire contact lens electrode (OcuScience) positioned on the corneal surface served as the recording electrode. Light flashes were delivered at intensities of 0.01, 0.1, 3, 6, and 25 cd·s/m^2^. For each flash intensity, ten ERG responses were recorded and averaged.

The a-wave amplitude was defined as the voltage difference between the baseline voltage measured immediately prior to flash onset and the most negative deflection following the stimulus. The b-wave amplitude was defined as the voltage difference between the a-wave trough and the subsequent maximal positive peak of the waveform. Longitudinal comparisons of ERG amplitudes were performed across time points and treatment groups using averaged responses from each animal. Repeated recordings obtained from the same subject at different post-treatment time points were treated as longitudinal measurements and were not considered independent biological replicates.

### Visually evoked potential (VEP) recording

VEP recordings were performed to evaluate cortical responses to visual stimulation. Mice were positioned in a stereotaxic frame, and the skull was exposed. A small craniotomy was performed to allow placement of the recording electrode over the primary visual cortex (V1). The recording electrode was positioned at approximately anteroposterior (AP) −3.0 mm and mediolateral (ML) +2.5 mm relative to bregma, corresponding to the contralateral visual cortex relative to the stimulated eye. A reference electrode was placed at AP +2.5 mm and ML −1.5 mm, and the ground electrode was inserted subcutaneously at the base of the tail.

### Optical coherence tomography imaging

Longitudinal retinal imaging was performed using spectral-domain optical coherence tomography (SD-OCT) to monitor potential structural changes in the retina following treatment compared with vehicle controls. Mice were anesthetized with a mixture of ketamine (65 mg/kg), xylazine (7.5 mg/kg), and acepromazine (0.5 mg/kg) and positioned on a maneuverable imaging platform to ensure stable alignment during imaging. Pupillary dilation was achieved by topical application of one to two drops of 1% tropicamide. During imaging, the corneal surface was maintained in a hydrated state using balanced salt solution to preserve optical clarity. SD-OCT imaging was performed using the NS-NEEL imaging system (Nanoscope Instruments). B-scan images of the retina were acquired at baseline and at subsequent time points following intravitreal injection of the treatment. For comparisons of retinal morphology before and after NOD, follow-up scans were aligned to the baseline scan using the optic nerve head and major retinal vessels as anatomical landmarks. Whenever possible, B-scans obtained from corresponding retinal locations were used for longitudinal comparisons. Retinal thickness measurements were obtained at multiple locations across the central retina to assess potential structural changes associated with treatment.

OCT B-scan images were analyzed using ImageJ software (National Institutes of Health). Approximately thirty B-scan images were collected per experiment. For consistency and to minimize measurement bias, thickness measurements were obtained at comparable retinal locations across all experimental groups. B-scan images in which the optic nerve head was positioned at the center of the scan were selected for analysis. The optic nerve head region was excluded from thickness measurements due to the absence of organized retinal lamination in this region, which would otherwise introduce variability in structural measurements. Multiple thickness measurements were obtained from each image and averaged to improve reproducibility.

### Intravitreal injection of MCO-020 and gold nanorods

All surgical procedures were performed using aseptic technique and sterilized surgical instruments. Mice were anesthetized by intraperitoneal injection of ketamine (90 mg/kg), xylazine (10 mg/kg), and acepromazine (0.5 mg/kg). Topical anesthesia was administered to the ocular surface using proparacaine hydrochloride ophthalmic solution.

Intravitreal injections were performed under a dissection microscope using a Hamilton microsyringe equipped with a 32-gauge needle. A total volume of 1 μL containing MCO-020 plasmid and gold nanorods was injected through the sclera into the vitreous cavity. Following injection, ciprofloxacin ophthalmic solution (0.3%) was applied to the eye to prevent infection. The cornea was maintained in a hydrated state throughout the procedure using balanced salt solution. The treatment formulations consisted of Dose 1, 0.5 μg/μL MCO-020 plasmid and 0.1 μg/μL GNR; Dose 2, 1.0 μg/μL MCO-020 plasmid and 0.1 μg/μL GNR; and Dose 3, 1.0 μg/μL MCO-020 plasmid and 0.2 μg/μL GNR. A total volume of 1 µL was administered into the treated eye.

### Nano-enhanced optical delivery (NOD)

After intravitreal injection of the plasmid–nanorod mixture, gene delivery was performed using nano-enhanced optical delivery (NOD). Prior to optical delivery, pupils were dilated using 1% tropicamide ophthalmic solution. The mouse was positioned under the optical delivery system, and the retina was visualized using OCT imaging. A near-infrared (NIR) laser beam was directed to the selected retinal region of interest. The wavelength of the NIR laser was chosen to match the surface plasmon resonance peak of the gold nanorods. Upon irradiation, the gold nanorods generated localized plasmonic heating that produced transient nanopores in the cell membrane. These transient membrane pores allowed uptake of extracellular plasmid DNA into retinal cells located within the irradiated region. Approximately 2 h after intravitreal injection, the treated eye was positioned under the OCT-guided delivery system. The retinal region of interest was identified using OCT en-face and B-scan imaging. A 980 nm continuous-wave NIR laser with an approximately 15 µm Gaussian focal spot was raster scanned over an approximately 1 × 1 mm retinal area. Laser powers of 10-30 mW and total irradiation durations of 200 and 400 s were evaluated. Retinal landmarks, including major blood vessels and the optic nerve head, were used to maintain alignment of the delivery beam with the selected target region throughout the procedure.

The principle of nano-enhanced optical delivery (NOD) is illustrated schematically in Figures 1C-F. In retinal degenerative diseases such as geographic atrophy, photoreceptors are lost while inner retinal neurons remain relatively preserved (Figure 1C). Following intravitreal injection, MCO-020 plasmids mixed with gold nanorods distribute within the retinal layers (Figure 1D). Targeted irradiation with a near-infrared (NIR) laser activates the gold nanorods through surface plasmon resonance, generating localized thermal hotspots at the cell surface (Figure 1E). This localized heating transiently permeabilizes the plasma membrane, allowing entry of extracellular plasmid DNA into cells within the irradiated region (Figure 1F). Once internalized, the MCO-020 gene is expressed in the targeted retinal neurons, rendering them photosensitive (Figure 1G). Activation of these modified neurons by light enables transmission of electrical signals through the retinal circuitry to higher visual centers, potentially restoring light responsiveness in regions of photoreceptor degeneration (Figure 1H).

### Cell culture for *in vitro* experiments

To evaluate the efficiency of nano-enhanced optical delivery (NOD) for plasmid transfer, initial experiments were performed in HEK293 cells. Cells were cultured in Dulbecco’s modified Eagle’s medium (DMEM/F-12) supplemented with 10% fetal bovine serum, 0.2 mg/mL streptomycin, and 200 U/mL penicillin and maintained at 37 °C in a humidified 5% CO_2_ atmosphere. Prior to NOD experiments, cells were incubated with gold nanorods (GNRs) and MCO-020 plasmids for 30 min. Targeted gene delivery was achieved by irradiating the cells with the OCT-guided continuous wave near-infrared (NIR) laser (980 nm).

### Immunostaining of cells and retinal tissue

HEK293 cells and retinal tissues were harvested and fixed in 4% paraformaldehyde in PBS overnight. After overnight fixation, samples were stored in 1X PBS until immunostaining was performed at 4 °C. For immunostaining, tissues were washed with PBS containing Triton X-100 to permeabilize the membranes. Nonspecific antibody binding was blocked using serum blocking solution prior to incubation with primary antibodies against the mCherry reporter protein. Following overnight incubation with the primary antibody (mCherry, NovusBio), samples were washed and incubated with fluorescent secondary antibodies (Alexa Fluor 594, Alexa Fluor 546). Nuclear staining was performed using DAPI. The stained retina samples were subsequently imaged using confocal microscopy.

### Confocal fluorescence imaging

Confocal fluorescence imaging was performed using a confocal microscope (Fluoview FV1000, Olympus). Images were acquired using excitation wavelengths corresponding to DAPI and fluorescent secondary antibodies. Confocal imaging was used to evaluate expression of the MCO-020 transgene and to assess cellular localization of the reporter protein. Image processing and quantitative analysis were performed using ImageJ software.

### OCT-guided NOD delivery system

The OCT-guided NOD system integrates retinal imaging with optical delivery to enable spatially targeted gene transfer (Figures 2A,B). The OCT subsystem uses a low-coherence superluminescent diode centered at 840 nm with a spectral bandwidth of approximately 60 nm. The OCT beam and the NIR delivery laser beam are combined using a fiber coupler so that both beams share a common optical path toward the retina.

**FIGURE 2:**
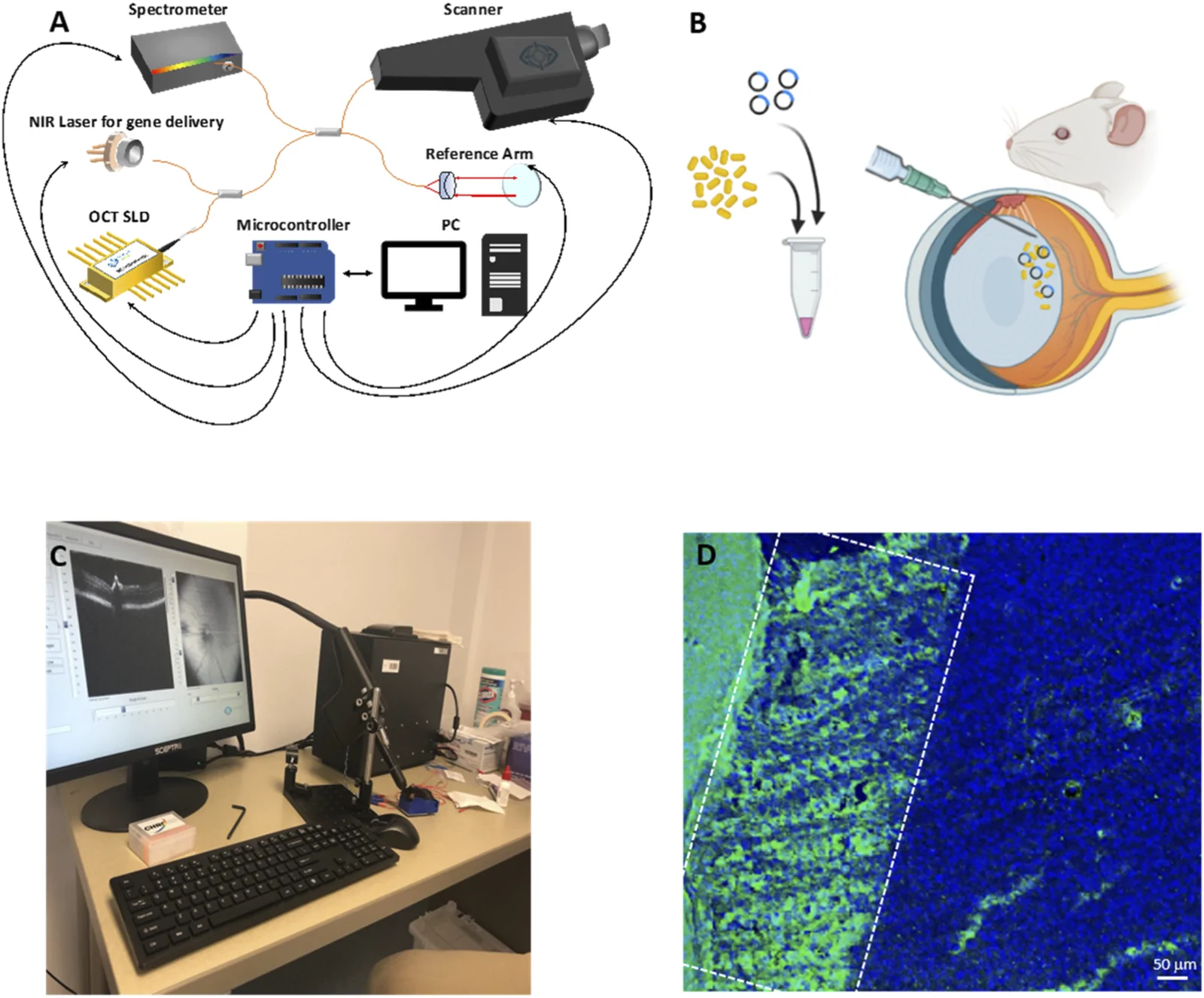
Schematic of OCT guided gene delivery platform. **(A)** Diagram showing OCT scanner head and Laser components; **(B)** Schematic of intravitreal injection of the MCO-020 plasmid-GNR mixture. **(C)** Experimental setup showing B-Scan and C-scan OCT image of mouse retina for selection of targeted region of interest for gene delivery. **(D)** Confocal fluorescence images of retinal explants showing expression of MCO-020-mCherry in the targeted area (as shown by the rectangular pattern in the immunostained image) 4 weeks after exposure to OCT-guided laser in the presence of GNR and MCO-020 plasmids.

The combined beam exiting the fiber splitter is collimated using a lens and directed toward the eye using micro-electromechanical system (MEMS) scanning mirrors and telescopic lenses. The reference beam is reflected from a stationary mirror and recombined with the backscattered light returning from retinal tissue. The resulting interference signal is detected by a spectrometer equipped with a line-scan camera. Fourier transformation of the detected spectrum produces depth-resolved structural OCT images of the retina. Using en-face OCT imaging (Figure 2C), the targeted region of interest for optical gene delivery is identified and marked on the imaging interface. The NIR laser is then directed to the selected region to facilitate localized gene delivery. Retinal features such as blood vessels were used as reference landmarks for tracking the position of the targeted region. This feedback system enabled dynamic adjustment of the delivery beam to ensure precise alignment with the selected retinal region during optical delivery. Figure 2D shows a confocal fluorescence image of a retinal explant with expression of MCO-020-mCherry in the targeted area.

### Retinal cell viability assessment

Retinal cell viability was assessed in a separate *in vivo* safety experiment using rd1 mice. The experimental conditions included untreated control, laser-only, GNR-only, and combined GNR-plus-laser treatment. Two weeks after treatment, retinas were isolated and dissociated into single-cell suspensions. Cell viability was quantified using the NC3000 automated cell-analysis system according to the manufacturer’s instructions. Each group included 3–4 mice and yielded approximately 4–8 × 10^6^ dissociated retinal cells for analysis. The combined treatment condition used the GNR and laser parameters selected during the preceding optimization experiments.

### *In Vivo* study design

The *in vivo* studies comprised several complementary experiments designed to evaluate delivery efficiency, dose dependence, durability, functional activity, and safety of OCT-guided NOD. In *rd1* mice, the effects of optical delivery parameters were evaluated using different laser power and irradiation-duration conditions, including 10 and 20 mW exposures for 200 or 400 s, with transgene expression assessed by retinal imaging and immunofluorescence (Figures 3, 4A,B). Separate formulation-dose experiments evaluated the effects of MCO-020 and GNR concentration on functional responses. The treatment formulations included combinations of 0.5 or 1.0 μg/μL MCO-020 with 0.1 or 0.2 μg/μL GNR (Figure 4C), while an additional GNR dose-response experiment evaluated 1.0 μg/μL MCO-020 in combination with increasing GNR concentrations for longitudinal ERG and VEP assessment (Figure 5). Longitudinal studies were used to evaluate persistence of transgene expression and electrophysiological responses over time. Safety was assessed separately using retinal cell-viability, intraocular pressure, OCT, ERG, and VEP measurements, including additional studies in wild-type mice (Supplementary Figures 2, 3). A separate focal laser-injury model was used to evaluate spatially targeted delivery and local functional responses in an experimentally defined region of retinal injury (Supplementary Figure 4). Sample sizes and experimental units for the individual experiments are provided in the corresponding figure legends.

**FIGURE 3:**
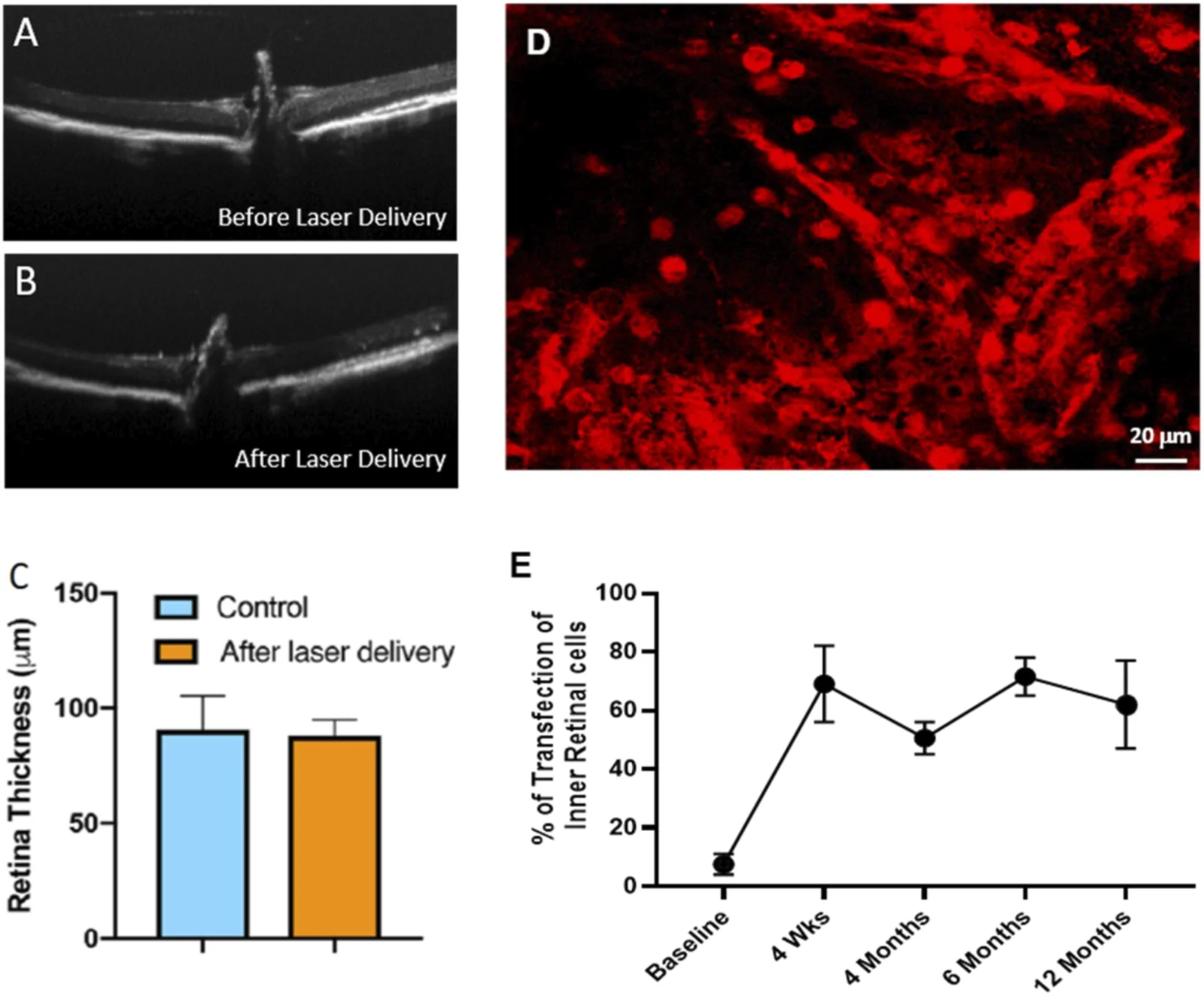
*In vivo* OCT-guided laser delivery of MCO-020. Expression of MCO-020 in mouse retina after OCT-guided laser gene delivery. **(A–B)** B-scan OCT image before and after laser irradiation, **(C)** quantification of retina thickness, **(D)** Cellular expression of MCO-020 (visualized by immunostained mCherry) in mouse retina. **(E)** Longitudinal quantification of retinal cells expressing MCO-020 transgene. Data are presented as mean ± SEM; n = 2/time-point.

**FIGURE 4:**
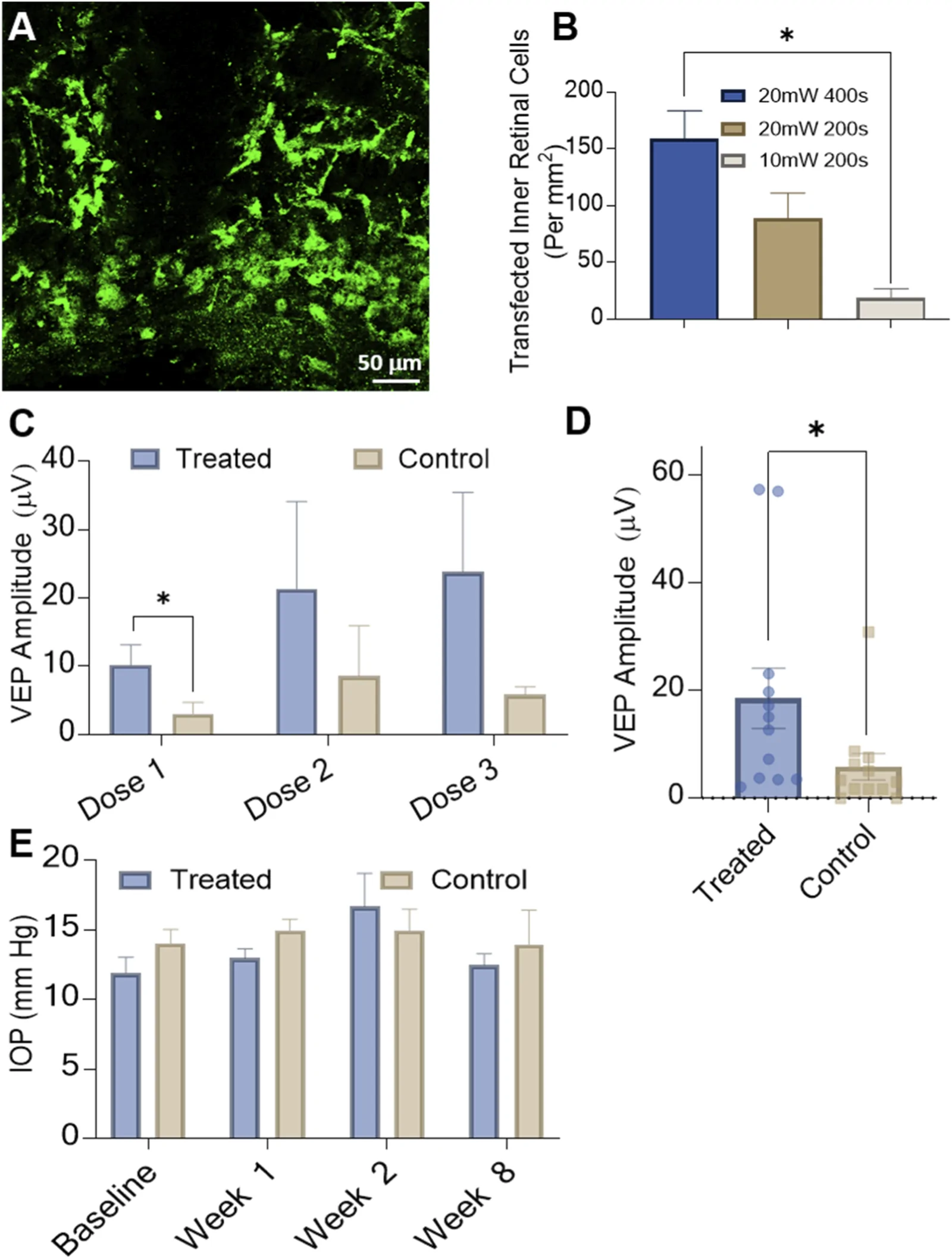
Robust *in-vivo* large-area delivery of MCO to inner retinal cells by OCT-guided optical delivery. **(A)** Confocal immunofluorescence images of mCherry (reporter) in the retinas of *rd1* mice 13 weeks after transfection by OCT-guided NIR (980 nm) laser (20 mW) based optical delivery of MCO-mCherry plasmids. **(B)** Number of RGCs expressing MCO-020 per mm^2^ in three different doses of laser, **(C)** Dose-dependent VEP measurements from visual cortex (V1 region) of MCO-020-mCherry treated mice and the control group. Dose 1: GNR 0.1 μg/μL, MCO-020 0.5 μg/μL, Dose 2: GNR 0.1 μg/μL, MCO-020 1.0 μg/μL, Dose 3: GNR 0.2 μg/μL, MCO-020 1.0 μg/μL. **(D)** VEP measurement from the visual cortex of *rd1* mice (responder group) after laser delivery of MCO-020-mCherry. **(E)** Laser-induced MCO-020 delivery does not alter intraocular pressure (IOP) in the injected *rd1* eyes. Data are presented as mean ± SEM; *n* = 4 per dose group; *p < 0.05.

**FIGURE 5:**
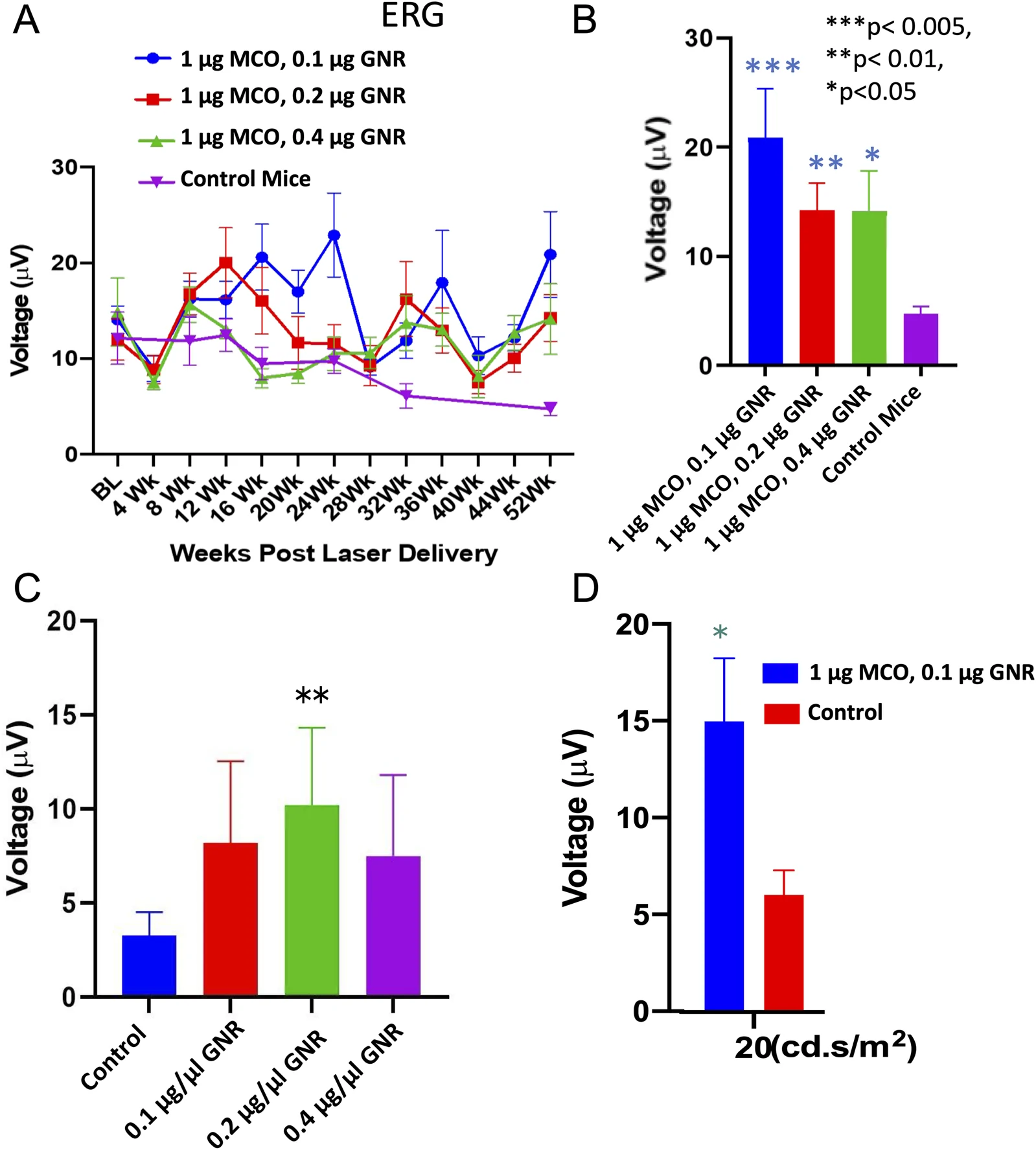
Enhanced ERG and VEP response in *rd1* mice treated with MCO-020. **(A)** Longitudinal ERG response in different conditions of NOD after MCO-020 injection. The control non-treated mice group is included. In the non-treated control group mice, ERG decreased over time, while for the treated groups, the ERG amplitude was maintained over time. **(B)** Quantification of ERG amplitude for different doses of GNR at the 52‐week time point shows significant improvement in ERG for the treated group compared to the non-treated control. **(C)** Improved VEP after MCO-020 treatment. *Rd1* mice with and without MCO treatment were subjected to flash VEP, and MCO-020-treated mice exhibited higher VEP amplitude compared to non-treated controls after 4 weeks. **(D)** The improved VEP amplitude was sustained through 52 weeks. Data are presented as mean ± SEM; *n* = 6 per dose group; *p < 0.05.

### MCO-020 plasmid biodistribution

Biodistribution of MCO-020 plasmid DNA was evaluated at 12 months following OCT-guided NOD delivery. Non-target tissues, including brain, gonads, kidney, mesentery, spleen, heart, lungs, and liver, were collected and analyzed for MCO-020 vector copy number. Vector copy numbers were expressed as copies per ng of DNA and compared with the limit of detection (LOD) of 50 copies/ng DNA.

### Statistical analysis

Statistical analysis was performed using GraphPad Prism software. Data are presented as mean ± standard deviation (SD) or mean ± standard error of the mean (SEM), as specified in the corresponding figure legends. The experimental unit and sample size for each analysis are provided in the corresponding figure legend. Repeated measurements obtained from the same subject were treated as longitudinal observations and were not considered independent biological replicates. Statistical tests were selected according to the experimental design and number of groups being compared. Comparisons involving multiple groups were analyzed with appropriate multiple-comparison procedures, and longitudinal datasets were analyzed using methods that accounted for repeated measurements within the same subject. The specific statistical test and correction used for each analysis are stated in the corresponding figure legends. A *p*-value <0.05 was considered statistically significant.

## Results

### *In vitro* validation of nano-enhanced optical delivery (NOD)

Immunostaining for the mCherry reporter protein revealed that MCO-020 transgene expression occurred only in HEK293 cells exposed to laser irradiation. In the absence of laser exposure, cells incubated with plasmid and GNRs showed no detectable mCherry expression (Figure 6A), confirming that laser activation is required for plasmid entry into cells. Laser-dependent transgene expression was further evident when comparing different irradiation parameters. Increasing laser power resulted in increased reporter expression, with cells irradiated at 20 mW exhibiting substantially stronger mCherry fluorescence compared with cells irradiated at 10 mW (Figure 6A). Similarly, longer irradiation durations resulted in higher levels of transgene expression. The region exposed to 20 mW scanning laser beam (total exposure for 400 s) showed the strongest reporter expression, whereas lower expression levels were observed at shorter exposure durations (20 mW for 200 s) or lower laser power (10 mW for 200 s).

**FIGURE 6:**
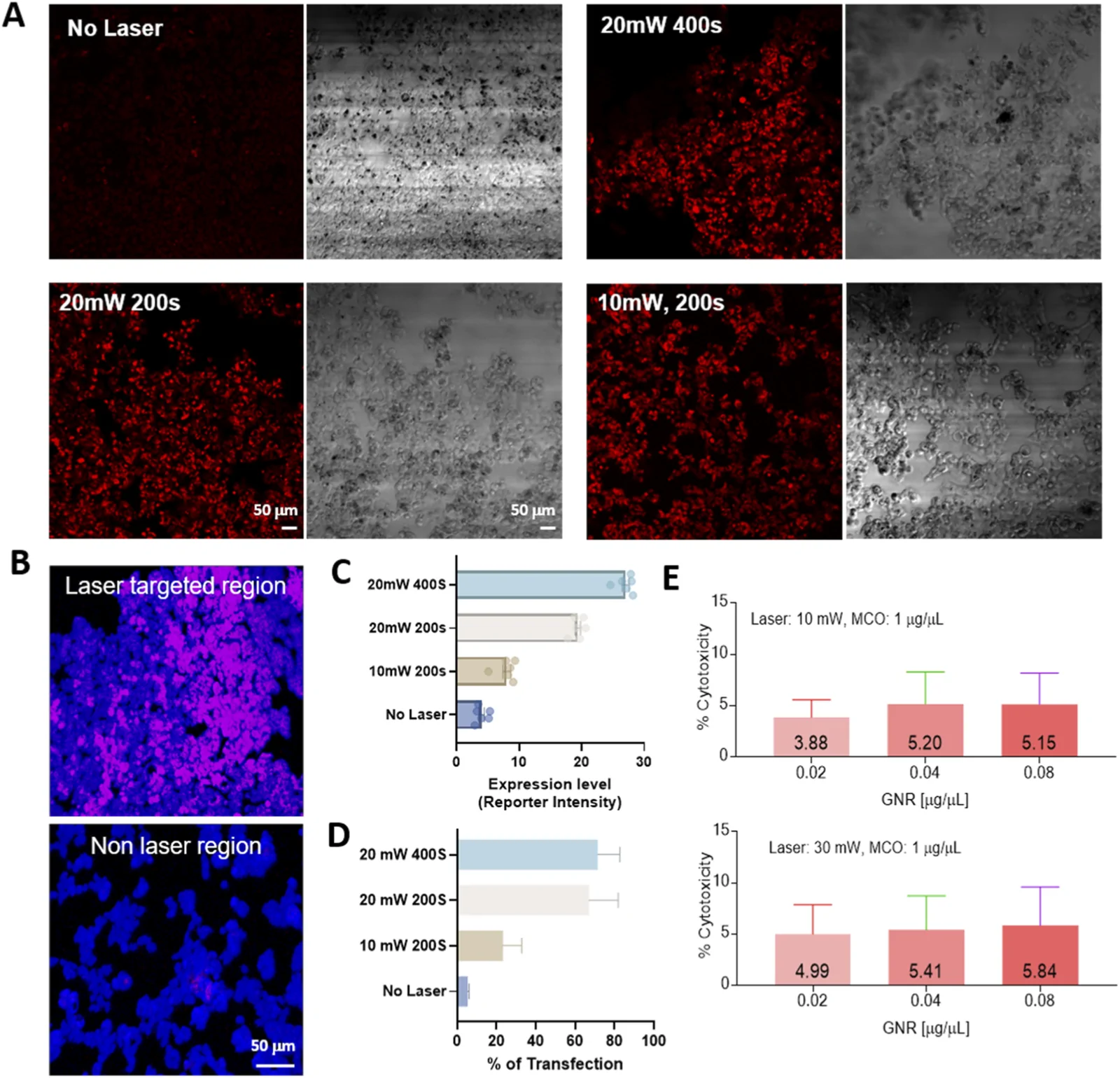
Dose‐dependent expression of MCO‐020‐mCherry in HEK 293 cells after targeted delivery using OCT-guided laser system. **(A)** Dose-dependent expression of MCO-020-mCherry in HEK cells. A gradual increase of transgene expression was evident upon the increase in laser power. **(B)** The laser causes expression of MCO-020-mCherry in the targeted region, but no expression in the non-targeted region. **(C)** Quantification of the expression of MCO-020-mCherry at different laser doses; and **(D)** Quantification of the transfection level of MCO-020-mCherry in different doses of the laser. Dose-dependent reporter expression was evident in human cells, *n* = 3 per dose group. **(E)** No increase in cytotoxicity in the GNR concentration range of 0.02–0.08 μg/μL. No dependence of cytotoxicity on Laser power in the 10–30 mW range.

The spatial selectivity of the NOD approach was further demonstrated by comparing laser-targeted and non-irradiated regions within the same culture. Strong mCherry expression was observed only in the laser-exposed region, while adjacent non-irradiated areas showed negligible reporter signal (Figure 6B), indicating that gene delivery was confined to the laser-targeted area. Quantitative analysis confirmed these observations, showing a clear dose-dependent increase in transgene expression with increasing laser power and irradiation duration (Figures 6C,D). These results demonstrate that NOD enables controllable and spatially targeted plasmid delivery by adjusting laser parameters. To evaluate the potential cytotoxicity of the NOD procedure, an MTT assay was performed following treatment with varying concentrations of GNRs and laser irradiation conditions. Cell viability remained unchanged across the tested GNR concentrations (0.02–0.08 μg/μL), and increasing the laser power from 10 mW to 30 mW did not result in detectable cytotoxicity (Figure 6E). These findings indicate that NOD-mediated plasmid delivery under the tested conditions is well tolerated *in vitro*.

### *In vivo* targeted retinal gene delivery using OCT-guided NOD

To assess the safety of OCT-guided NOD delivery, retinal morphology was monitored using optical coherence tomography (OCT) before and after laser irradiation. Representative B-scan OCT images acquired prior to treatment showed the characteristic retinal morphology of *rd1* mice, including a marked loss of the outer retina consistent with photoreceptor degeneration (Figure 3A). OCT imaging performed immediately following laser irradiation revealed no detectable disruption of retinal structure or evidence of additional tissue damage within the targeted region (Figure 3B). Retinal thickness measurements obtained from OCT B-scan images showed no statistically significant differences between baseline and post-treatment measurements (Figure 3C), indicating that the overall retinal structure remained stable following OCT-guided NOD delivery.

Successful transgene delivery was confirmed by immunofluorescence staining of retinal tissues. Confocal microscopy demonstrated robust expression of the MCO-020 transgene, visualized through immunostaining of the mCherry reporter, in retinal cells located within the laser-targeted region (Figure 3D).

Longitudinal quantification of mCherry-positive retinal cells revealed persistent expression of the MCO-020 transgene for several months following delivery (Figure 3E). Although the 12-month data point represents a partial dataset, detectable transgene expression remained evident, suggesting long-term stability of the delivered plasmid.

### Robust expression of MCO-020 in inner retinal cells

To further characterize the distribution of transgene expression following delivery, retinal tissues from treated *rd1* mice were examined using confocal immunofluorescence microscopy. Strong mCherry reporter expression was observed in the inner retinal cells within the laser-irradiated regions of the retina (Figure 4A). Quantitative analysis showed that the number of MCO-expressing cells increased with increasing laser power (Figure 4B), indicating that higher laser power resulted in greater transfection efficiency.

### Functional restoration of visual responses following MCO-020 delivery

To evaluate the functional consequences of MCO-020 expression in the degenerative retina, electrophysiological recordings were performed in *rd1* mice. Visually evoked potential (VEP) recordings were obtained from the visual cortex to determine whether retinal activation following MCO-020 delivery could propagate to higher visual centers. Dose-dependent increases in VEP responses were observed following MCO-020 delivery (Figure 5C). In animals that exhibited strong retinal expression of the transgene, VEP responses were clearly detectable in the visual cortex (Figure 5D), confirming successful transmission of visual signals through the retinal circuitry. Untreated *rd1* mice exhibited low residual cortical responses under the recording conditions used. MCO-020-treated mice showed significantly greater VEP amplitudes than untreated controls, with mean responses of approximately 15 µV and 6 μV, respectively (p < 0.05), indicating a treatment-associated increase above the *rd1* control background. In addition, intraocular pressure (IOP) measurements obtained from treated animals showed no significant changes compared with control eyes (Figure 4E), suggesting that the delivery procedure does not adversely affect ocular physiology.

### Safety and toxicity assessment of NOD-mediated gene delivery

To assess potential retinal toxicity associated with NOD delivery, retinal cell viability was evaluated following treatment. Dissociated retinal cells from treated *rd1* mice were analyzed 2 weeks after intravitreal injection and laser irradiation (Supplementary Figure 2A). Quantitative analysis showed no significant differences in cell viability between untreated controls, laser-only treated animals, GNR-only treated animals, and animals receiving combined GNR and laser treatment (Supplementary Figure 2B). The minor variability observed between groups was attributed to experimental variation during tissue dissociation. Longitudinal monitoring of intraocular pressure further confirmed the ocular safety of the treatment. IOP measurements obtained from MCO-020-treated mice remained comparable to those from untreated control animals throughout the study period (Supplementary Figure 2C). Additional safety assessments were performed in wild-type mice. ERG recordings showed no deleterious effects on retinal function following treatment (Supplementary Figure 3A). OCT imaging revealed no significant differences in retinal thickness between treated and control animals (Supplementary Figure 3B). Similarly, VEP recordings demonstrated no impairment of cortical visual responses following treatment (Supplementary Figure 3C). Biodistribution analysis performed at 12 months showed below LOD MCO-020 vector DNA in the evaluated non-target tissues, including brain, gonads, kidney, mesentery, spleen, heart, lungs, and liver (Supplementary Figure 5). Vector copy numbers in all non-target tissues remained below the predefined reference threshold of 50 copies/ng DNA and were comparable to control levels, supporting limited systemic distribution following ocular delivery.

### Local restoration of retinal function in a laser injury model

To further evaluate the therapeutic potential of the NOD in localized retinal degeneration, a mouse model of laser injury was generated by inducing focal photoreceptor injury using a green laser beam. OCT en-face imaging confirmed localized retinal damage within the targeted region (Supplementary Figure 4A,B). Microfocal ERG recordings obtained from the damaged area showed markedly reduced responses prior to treatment. Following OCT-guided NOD delivery of MCO-020, significant recovery of focal ERG responses was observed within 1 week (Supplementary Figure 4C), demonstrating restoration of localized retinal activity.

## Discussion

Retinal degenerative diseases such as geographic atrophy are characterized by progressive loss of photoreceptors, while inner retinal neurons often remain structurally preserved. This preservation provides an opportunity to restore visual responses by targeting these surviving neurons. Optogenetic therapy has therefore emerged as a promising strategy for vision restoration by introducing light-sensitive proteins into inner retinal neurons to re-establish light responsiveness. In the present study, we demonstrated that NOD enables targeted non-viral delivery of the MCO-020 opsin gene into retinal neurons using OCT-guided continuous wave near-infrared laser irradiation. *In vitro* viability assays showed no detectable cytotoxicity across the tested range of laser powers and gold nanorod concentrations, supporting the safety of the delivery conditions used in this study. These *in-vitro* experiments in HEK293 cells were conducted as short-term studies to establish the feasibility and efficiency of laser-mediated plasmid delivery and to optimize the delivery parameters.

*In vivo* experiments further demonstrated that OCT-guided NOD enables targeted delivery of the MCO-020 plasmid to retinal neurons while preserving retinal structure. Similar to that in electroporation, lipofection, or gene gun delivery, once released into the cytoplasm, the naked plasmid binds to various cytoplasmic proteins and transcription factors. These DNA-binding proteins interact with molecular motor proteins that carry the plasmid cargo and move actively along the microtubule network toward the nuclear envelope. In the non-dividing post-mitotic neurons, the importin-protein-plasmid complex docks onto the nuclear pore complex and is actively ferried into the nucleoplasm. Immunofluorescence imaging confirmed successful transgene expression in retinal cells within the laser-targeted regions, and longitudinal analysis demonstrated persistent MCO-020 expression. The ability to achieve sustained transgene expression using a non-viral delivery approach is particularly important for therapeutic applications.

Although plasmid-mediated expression is generally considered transient, plasmid DNA can persist for prolonged periods in postmitotic tissues, where loss through cell division is limited (Jiao et al., 1993; Gasiorowski and Dean, 2005). Importantly, VEP recordings obtained from the visual cortex demonstrated that retinal activation following MCO-020 expression produced measurable cortical responses, confirming the successful propagation of visual signals through the visual circuitry. The electrophysiological findings demonstrate that OCT-guided NOD delivery of MCO-020 generated sustained light-evoked activity at both the retinal and visual-cortical levels, supporting objective evaluation of functional improvements at local as well as higher-order visual centers in a mouse model of complete photoreceptor degeneration. The current study specifically establishes the feasibility of spatially targeted delivery by the OCT-guided non-viral delivery platform, along with durability of expression and efficacy of non-virally delivered MCO-020, measured by objective electrophysiological assessments.

Electrophysiological measurements further demonstrated that MCO-020 expression leads to improved retinal and cortical responses in the completely photoreceptor-degenerated retina. It is important to note that *rd1* mice undergo rapid photoreceptor degeneration and typically exhibit an absence of ERG responses. Expression of the opsin in surviving retinal neurons enables direct light sensitivity in these cells, allowing them to generate electrical responses. Consistent with this mechanism, animals receiving MCO-020 delivery exhibited enhanced ERG responses compared with untreated controls (Figure 5), while visually evoked potential recordings demonstrated significantly increased cortical responses. These cortical responses persisted for up to 52 weeks following treatment, indicating long-term functional activity of the optogenetically modified retinal neurons. Conventional ERG a- and b-waves primarily reflect photoreceptor and bipolar-cell activity, respectively, and are markedly diminished or absent in *rd1* mice (Bhatt et al., 2023). Therefore, the light-evoked corneal potentials detected following MCO-020 delivery should not be interpreted as recovery of normal outer-retinal function. MCO-020 expression can generate direct light-evoked ionic currents in surviving inner retinal cells, which may contribute to the recorded ERG signal. *In the case of AAV-based delivery of MCO, ERG response has also been reported (* Batabyal et al., 2024 *)*.

Safety evaluation is critical for the translational development of new gene delivery platforms. Retinal inflammatory responses associated with the NOD platform were evaluated previously using Iba1 immunostaining for microglial activation, with no detectable NOD-associated increase in activated microglia under the tested conditions (Batabyal et al., 2019). The present OCT-guided NOD study is focused on complementary safety endpoints, including OCT-based retinal structure, retinal-cell viability, IOP, and electrophysiological function. Retinal cell viability assays showed no detectable differences in cell survival following laser irradiation or nanoparticle exposure (Supplementary Figure 2). Longitudinal monitoring of intraocular pressure revealed no changes following treatment, indicating that the procedure does not adversely affect ocular physiology. Additional studies performed in wild-type mice showed no detectable alterations in ERG responses, OCT-measured retinal thickness, or VEP responses after NOD delivery, further confirming the absence of deleterious functional or structural effects (Supplementary Figure 3). One of the key advantages of the NOD platform is its ability to achieve spatially targeted gene delivery. For retinal diseases such as geographic atrophy in advanced AMD, degeneration occurs in localized retinal regions rather than uniformly across the retina. The OCT guidance enables the identification of these degenerated regions and thus, targeted delivery of therapeutic genes could be achieved directly to the affected areas. This capability was further demonstrated in a focal photoreceptor injury model mimicking geographic atrophy, where localized NOD delivery of MCO-020 resulted in recovery of microfocal ERG responses in damaged retinal regions (Supplementary Figure 4). Direct retinal temperature measurements and GNR-specific thermal modeling were not performed in this study. The reported exposure calculations therefore characterize the delivered optical dose but do not establish the local temperature rise at the GNR–cell interface. Nevertheless, the absence of detectable changes in retinal morphology, thickness, cell viability, IOP, and electrophysiological function supports tolerability within the tested operating range.

The ocular retention of related functionalized GNRs used in this delivery platform was evaluated previously using longitudinal OCT, fluorescence imaging, scanning electron microscopy, and energy-dispersive X-ray analysis. Detectable retinal GNR association declined substantially after approximately 1 week following intravitreal administration (Dhakal et al., 2015; Batabyal et al., 2021; Batabyal et al., 2019). The present study therefore focused on complementary safety endpoints, including OCT-based retinal structure, retinal-cell viability, IOP, and electrophysiological function. A limitation of the present study is that the full therapeutic window of OCT-guided NOD was not defined by escalation from ineffective exposure to the threshold for tissue injury. Although the tested laser and GNR conditions produced dose-dependent transgene expression without detectable changes in cell viability, retinal thickness, IOP, or electrophysiological function, these findings establish safety only within the evaluated operating range. Published retinal laser-damage thresholds provide general guidance but may not directly predict the threshold for NOD because plasmonic GNRs locally enhance optical absorption and heat generation.

Compared with viral gene delivery approaches, the NOD platform offers several potential advantages. Viral vectors such as AAV have limited packaging capacity and may induce immune responses or inflammatory reactions. In contrast, NOD enables delivery of plasmid DNA without the use of viral vectors, eliminating risks associated with viral integration and immune activation. Additionally, NOD allows delivery of larger genetic payloads, including gene-editing systems and other therapeutic constructs that exceed the packaging limits of viral vectors. Another important advantage of NOD is its integration with OCT imaging, which enables real-time visualization of retinal structures and precise targeting of delivery sites. This imaging-guided approach allows the therapeutic intervention to be directed specifically to diseased retinal regions while minimizing exposure of healthy tissue. The combination of OCT guidance and nano-enhanced optical delivery, therefore, represents a powerful strategy for achieving controlled and localized gene therapy. The ability to achieve sustained transgene expression, restore visually evoked responses, and maintain retinal structural integrity highlights the therapeutic potential of this approach. These findings support further development of OCT-guided NOD for the treatment of retinal degenerative diseases, including geographic atrophy.

## Conclusion

In this study, we demonstrate that OCT-guided nano-enhanced optical delivery (NOD) enables spatially targeted, non-viral delivery of the MCO-020 gene to retinal neurons using plasmonic gold nanorods and near-infrared laser irradiation. The integration of OCT imaging allows real-time guidance of gene delivery to selected retinal regions while preserving retinal structure. NOD-mediated delivery produced sustained MCO-020 expression and improved retinal and cortical electrophysiological responses in degenerative retina without detectable toxicity. Safety evaluations using OCT imaging and histological analysis showed no detectable retinal damage associated with laser-mediated gene delivery. In addition, immunostaining studies revealed no evidence of cytotoxicity in retinal cells expressing the MCO-020 transgene. These findings support the potential of OCT-guided NOD as a minimally invasive platform for targeted retinal gene therapy. Beyond optogenetic therapy, this approach may enable delivery of other therapeutic genes and gene-editing tools for the treatment of retinal degenerative diseases, including geographic atrophy.

## Data Availability

The original contributions presented in the study are included in the article/Supplementary Material. Further inquiries can be directed to the corresponding author.
