# Supplementary material for: Optical coherence tomography guided targeted non-viral optical delivery of opsin-encoding genes into the retina
Source: Front Pharmacol. 2026 Oct 19;17:1831339. doi: 10.3389/fphar.2026.1831339 (PMC13724445; doi:10.3389/fphar.2026.1831339)
Supplement: Supplementary file 1 [file Supplementaryfile1.docx]

**Supplementary Information**

**Optical Coherence Tomography guided targeted non-viral optical delivery of opsin-encoding genes into the retina**

Sanghoon Kim1, Michael Carlson1, Subrata Batabyal1, Adnan Dibas1, Samarendra Mohanty1,2*

^1^Nanoscope Technologies LLC., 1312 Brown Trail, Bedford, Texas 76022

^2^Nanoscope Therapeutics, Inc., 2777 N. Stemmons Fwy, Dallas, Texas 75207

*Corresponding author

Email: [smohanty@nanoscopetech.com](mailto:smohanty@nanoscopetech.com)

**Supplementary Figure 1. Characterization of gold nanorod dimensions and optical absorbance.**
Size-distribution histogram of GNR width measured from TEM images (*n* = 75 nanorods), (A) with an average width of approximately 25 nm and a standard deviation of 6.5%, and (B) with an average length of approximately 120 nm and a standard deviation of 10.5%. (C) UV–Vis–NIR absorbance spectrum of the GNR formulation showing the plasmonic absorption band in the near-infrared region, overlapping with the 980 nm wavelength used for NOD.

**Supplementary Figure 2. Evaluation of retinal cell viability in *rd1* mice after laser gene delivery.** (A) Steps used for the preparation of retinal cells used viability assay. (B) Quantification of retinal cell viability (NC3000 system) after laser/GNR treatment. N = 4-8E6 cells/group, 3-4 mice/group. (C) Longitudinal monitoring of IOP measured in *rd1* mice (*n* = 6/group) after MCO-020 treatment. The control non-injected mouse group is also included.

**Supplementary Figure 3**. **Structural and functional toxicity assessment in wild-type and *rd1* mice after laser gene delivery measured by ERG, OCT, and VEP in wild type mice.** (A) ERG in wild-type (WT) mice treated with MCO-020 (*n* = 6/group). No deleterious effect on ERG was observed in WT mice subjected to laser gene delivery. Av. ± SEM. (B) Measurement of OCT thickness in *rd1* mice at 52 weeks. Retinal thickness was measured from OCT B-scan images in *rd1* mice (*n* = 6/group) treated with MCO-020 in the presence of different GNR doses. Avg. ± SEM. (C) Visually evoked potential (VEP) in WT mice (*n* = 4/group) treated with MCO-020. No deleterious effect on VEP was observed in WT mice subjected to laser gene delivery. Av. ± SEM.

**Supplementary Figure 4. Targeted optical delivery of MCO led to improved microfocal ERG in mouse models for dry-AMD.** OCT en face image of (A) normal mouse retina, and (B) after local photoreceptor damage. Scotopic μfERG from a targeted photoreceptor-damaged region showed diminished responses and improved μfERG response 1 week after OCT-guided optical delivery of MCO to the photoreceptor-damaged retina region. (C) B-wave magnitude from treated (OD of MCO in laser-injured) and non-treated (laser-injured) retinal region. Av. ± S.D., *p<0.05.

**Supplementary Figure 5. Biodistribution of MCO-020 plasmid at 12 months following OCT-guided NOD delivery.** MCO-020 vector copy number was quantified in non-target tissues, including brain, gonads, kidney, mesentery, spleen, heart, lungs, and liver, from mice receiving the MCO-020/GNR formulations and from untreated controls. Vector copy numbers in all evaluated non-target tissues remained below the 50 copies/ng DNA (LOD) indicated by the dashed red line. Data are presented as mean ± SEM.
